# Glutamate Scavenging as a Neuroreparative Strategy in Ischemic Stroke

**DOI:** 10.3389/fphar.2022.866738

**Published:** 2022-03-23

**Authors:** Oykum Kaplan-Arabaci, Alperen Acari, Pinar Ciftci, Devrim Gozuacik

**Affiliations:** ^1^ Koç University Research Center for Translational Medicine (KUTTAM), Istanbul, Turkey; ^2^ Sabancı University Nanotechnology Research and Application Center (SUNUM), Istanbul, Turkey; ^3^ School of Medicine, Koç University, Istanbul, Turkey

**Keywords:** stroke, glutamate, neuroreparation, excitotoxicity, neuroprotection, ischemia, TPA, brain

## Abstract

Stroke is the second highest reason of death in the world and the leading cause of disability. The ischemic stroke makes up the majority of stroke cases that occur due to the blockage of blood vessels. Therapeutic applications for ischemic stroke include thrombolytic treatments that are in limited usage and only applicable to less than 10% of the total stroke patients, but there are promising new approaches. The main cause of ischemic neuronal death is glutamate excitotoxicity. There have been multiple studies focusing on neuroprotection via reduction of glutamate both in ischemic stroke and other neurodegenerative diseases that ultimately failed due to the obstacles in delivery. At that point, systemic glutamate grabbing, or scavenging is an ingenious way of decreasing glutamate levels upon ischemic stroke. The main advantage of this new therapeutic method is the scavengers working in the circulating blood so that there is no interference with the natural brain neurophysiology. In this review, we explain the molecular mechanisms of ischemic stroke, provide brief information about existing drugs and approaches, and present novel systemic glutamate scavenging methods. This review hopefully will elucidate the potential usage of the introduced therapeutic approaches in stroke patients.

## Introduction

Stroke is the second leading cause of death after ischemic heart disease and the third cause of disability affecting more than six million lives per year, according to the latest report of the World Health Organization (WHO) (WHO, 2020). Stroke patients are broadly divided into ischemic and haemorrhagic stroke types while the majority has been driven by ischemia. The summation of damage done by both stroke types affect the life of 13,7 million people and cause 5,5 million deaths every year (Global GBD 2016 Stroke Collaborators, 2019). Considering the continuous increase in the human population as well as skyrocketing numbers in obesity and hypertension patients, it is likely to face severe circumstances in the upcoming years.

### Ischemic Stroke

The word ischemia is originated from two Greek words: ischō, meaning holding back and haima, meaning blood; expressing the phrase of stopping blood (Verdouw et al., 1998). As the name indicates, ischemic stroke simply occurs due to the interruption of blood supply to the brain. The human brain requires constant blood flow providing oxygen and nutrients as well as eliminating carbon dioxide and cell debris. When there is a disruption in the blood supply, the brain cells lose their only energy source glucose and eventually die because of the energy shortage combined with toxic waste that is accumulated in the cells. The ischemic brain could be classified into two regions based on the stroke’s severity: the core zone which lost 90% of blood flow and shows the most severe effects, and the penumbra or peri-infarct region which surrounds the core zone (Appireddy et al., 2015). Even though the core zone experiences partial cell death as ischemia happens, the circulation in that region partially continues by the collateral arteries and the damage is reversible if the blood flow could be restored within a couple of hours (Heiss and Rosner, 1983). Consequently, the penumbra or peri-infarct region has been the primary target of pharmacological approaches with the hope to rescue invaluable neuronal cells from death (Castillo et al., 2016).

It is momentous to emphasize that more focused attempts for the prevention and treatment of ischemic stroke are desperately required. Modern treatment methods are simply dependent on re-sustaining blood flow as soon as possible and ensuring reperfusion in the affected area. Current clinical therapies offer only two efficient methods: intravenous administration of tissue plasminogen activator (tPA) and mechanical thrombectomy (Bhaskar et al., 2018). Globally, tPA application is the most widely accepted and implemented treatment method for ischemic stroke, although it is only effective when quickly applied in the time window of 4.5 h following the initiation of stroke (Yoo et al., 2011), and in some cases it can go up to 6 h (Snelling et al., 2019). In addition to the narrow time window, there is a certain risk of reperfusion damage that makes the treatment capacity of tPA limited on most patients (Kurth et al., 2007). If the size of the clot occluding the vein is large than the tPA treatment limits or the time window is outlasted, MT surgery is the alternative treatment in which a microcatheter is used to directly remove the clot, although it is a risk involved solution that is far from being perfect (Dong et al., 2020). Despite the fact that there are hundreds of treatment attempts for thrombectomy, they ultimately failed to translate to the clinics (Matei et al., 2021). Considering all modern applications, it is safe to say there is a desperate need for pharmacological therapeutics and drugs that could solve the urgent need of ischemic stroke patients.

### Cellular Mechanism of Ischemia

**FIGURE 1:**
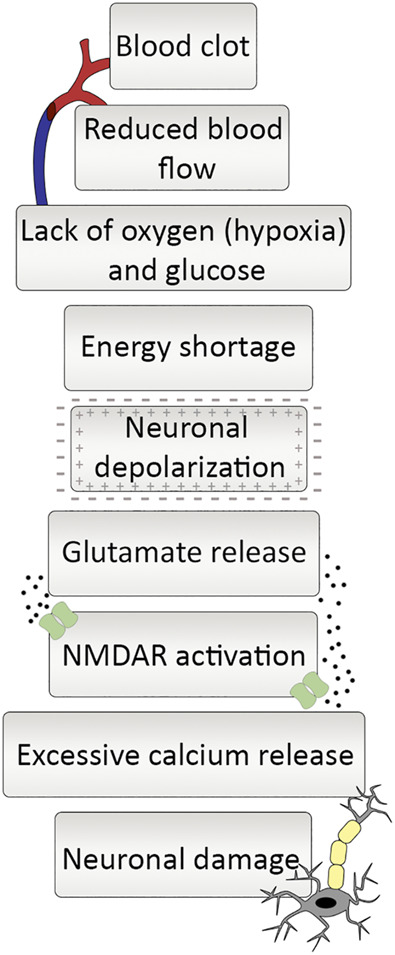
Ischemic cascade at a glance. Occlusion of an artery starts a series of responses. Blood clot blocking artery causes reduced blood flow, which leads to lack of oxygen and glucose and ultimately a drastic decrease in energy supply. It leaves neurons in a depolarized state and reduction in glutamate reuptake gives rise to increase in extracellular glutamate levels. The increment in glutamate leads to NMDA receptor activation and neuronal calcium influx, and excessive amount of calcium release ending up with neuronal damage.

The mechanism of ischemic cell death in the brain is well-known at the cellular and molecular levels (Rossi et al., 2007). A clot blocking an artery disrupts the consistent flow of nutrient-rich blood. Inevitably, it causes inadequate glucose and oxygen supply that cannot meet the energy demands of neuronal cells. Since glucose-dependent oxidative phosphorylation sourced by the constant blood flow is the primary energy source of neural cells, inadequacy in the glucose supply causes ATP depletion even in minutes because of the absence of long-term energy reserves in the brain (Doyle et al., 2008). Neuronal cells cannot perform regular metabolic activities without glucose and oxygen, causing inevitable starvation-related cell death (Figure 1). A second emerging problem arises with energy scarcity. The function of ion pumps is essential for the information transfer between the adjacent neurons that maintain the concentration gradient of sodium (Na^+^), potassium (K^+^), and calcium (Ca^2+^) between intracellular and extracellular environments but require ATP to function. The ion pumps are non-functional without energy, making the electrochemical gradient difference unsustainable. In the ATP depletion, the abnormal increase of Na^+^ influx and K^+^ efflux to neural cells causes depolarization. Following extracellular K^+^ concentration increase, L-type voltage-gated channels open and allow the passage of Ca^2+^ ions resulting in elevated intracellular Ca^2+^ concentration (Luoma et al., 2011). The Ca^2+^ cannot be pumped out of the cell by the ion pumps and exchangers without sufficient energy. The level of Ca^2+^ causes the release of glutamate, which is one of the fundamental reasons for ischemia-induced excitotoxicity in neurons and glial cells. Glutamate is an excitatory amino acid and behaves as a neurotransmitter binding glutamate receptor to transmit signals. Glutamate is released by the activation of its postsynaptic receptors that are ionotropic or metabotropic receptors (mGluRs). Ionotropic receptors consist of three groups which are NMDA (N-methyl-D-aspartate), AMPA (a-amino-3-hydroxy-5-methyl-4-iso-xazolepropionic acid) or kainate receptors (Atoji and Sarkar, 2019). Metabotropic receptors act through the heterotrimeric guanine nucleotide-binding proteins that convey the signal to its effector channels or intracellular enzymes. The excitotoxic response is primarily regulated by NMDA type glutamate receptors (Gupta et al., 2013; Girling et al., 2018).

The accumulation of glutamate in the extracellular space led to the hyperactivation of glutamate receptors and then to massive Ca^2+^ influx. If there is sufficient energy supply, ion equilibrium of the cells are maintained by removing some positive ions *via* ion pumps and then uptake the cell. However, in the case of insufficient energy in the cell, the ion pumps do not function properly, causing an important increase in the intracellular Ca^2+^. This intracellular Ca^2+^ increase causes the activation of protein kinases and other downstream Ca^2+^-dependent enzymes which damage significant molecules and disrupt the cell membrane, resulting in more Ca^2+^ entry into the cell, release of free radicals from damaged mitochondria, and subsequent cell death (Papazian et al., 2018; Verma et al., 2018; Belov Kirdajova et al., 2020).

**FIGURE 2:**
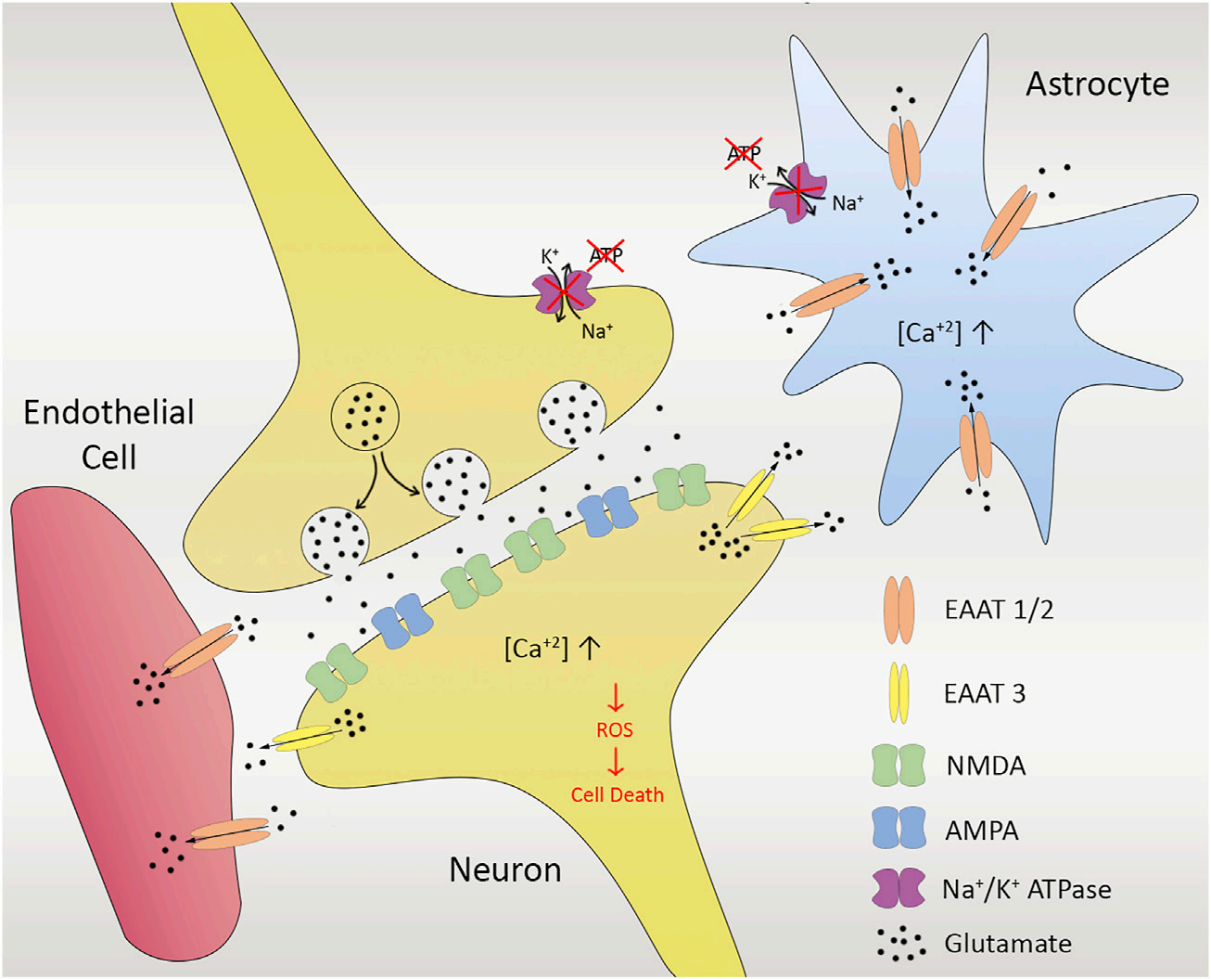
Ischemia in cellular unit. Anoxic depolarization and decreased activity of glutamate uptake gives rise to enhanced glutamate levels, leading to calcium influx *via* NMDA receptors. Excessive glutamate released from presynapse of the neuron also leads to Na^+^-dependent excitatory amino acid transporters (EAATs) in astrocytes (EAAT1/2) as well as neurons (EAAT3). Insufficient energy and lack of ion pump functioning cause massive increase in the intracellular Ca^2+^, activating protein kinases and some other downstream Ca^2+^-dependent enzymes, damaging the cell membrane, resulting in more Ca^2+^entry into the cell, release of free radicals as well as reactive oxygen species (ROS) that gives rise to cell death in the end.

The uptake of excessive glutamate by astrocytes also causes constant activation of Ca^2+^ channels that give rise to a pathological increase in intracellular Ca^2+^ concentration. Toxic elements such as reactive oxygen species (ROS) and a group of Ca^2+^-dependent degradative enzymes such as ATPases, endonucleases, proteases, and phospholipases are released from the neural cells (Sattler et al., 1999). Overall, the sequence of events results in the release of mitochondrial apoptotic signals and initiation of the caspase-dependent cell death (Belov Kirdajova et al., 2020) (Figure 2). The removal of excitatory neurotransmitters released to the synaptic cleft is also an energy-dependent action. Therefore, there is a huge increase in neurotransmitter concentrations, especially glutamate, in the ischemia, which acts as a mediator of neuronal degeneration.

### Glutamate and its Excitotoxicity

Glutamate is one of the most abundant amino acids in the human body participating in multiple metabolic pathways, and it is the most indispensable regulator in the central nervous system playing a vital role in regulating neural communication, plasticity, learning, memory, and more. Considering its broad range of metabolic activities and regulatory capacity, it is no wonder that glutamate-including pathways are highly regulated. The neurotransmitter function of glutamate requires its presence in the extracellular fluid part of the brain. Neuronal cells are sensitive to the changes in the extracellular glutamate level, where sustaining the balance is vital; therefore, the extracellular glutamate is regulated by release and restrain cycles. If the balance in the glutamate level is disturbed and the extracellular glutamate concentration increases in an uncontrollable manner, neuronal cells die inevitably because of the cumulative toxic effects (Zauner et al., 1996). This phenomenon is described as glutamate excitotoxicity and is known as a primary damaging mechanism that causes the cell death occured in stroke (Danbolt, 2001).

There are several mechanisms that supply glutamate to the extracellular fluid. The synaptic glutamate released from the presynapse is the main source of extracellular glutamate (Danbolt, 2001). The transfer of information at synapse requires receptor activation on postsynaptic side by sensing glutamate. As the postsynaptic receptors are outwardly located on the cell membrane, glutamate should be released from the presynapse to the extracellular fluid via exocytosis of the synaptic vesicles. Besides, there are several non-vesicular mechanisms that supply glutamate to the extracellular fluid via anion channels and reversed operation of glutamate transporting proteins (Rossi et al., 2000). Once released, the extra glutamate should be eliminated rapidly to sustain the balance. There is no mechanism to remove extracellular glutamate besides cellular reuptake by astrocytes, neurons and endothelial cells. The cellular reuptake is strictly controlled by Na^+^-dependent excitatory amino acid transporter family members, also known as excitatory amino acid transporters (EAATs) (Danbolt 2001; Grewer and Rauen, 2005; Tzingounis and Wadiche, 2007; Vandenberg and Ryan, 2013).

EAATs, which are a part of the solute carrier 1 (SLC1) family that uptake glutamate inside to the cell against the concentration gradient. There are five distinct types of these glutamate transporters. EAAT1 is predominantly expressed in the neocortex and cerebellum, specifically in astrocytes, whereas EAAT2 is the primary glutamate transporter found in the forebrain, widely expressed in astrocytes (Danbolt et al., 1990; Pines et al., 1992; Storck et al., 1992). Given that EAAT1 and EAAT2 have a broad expression in astrocytes, EAAT3 is mostly expressed in neurons (Rothstein et al., 1994; Mennerick et al., 1998). EAAT4 is also shown to be expressed in neurons, however the expression is limited to Purkinje cells. EAAT5 is on the other hand, only expressed at photoreceptor and bipolar cell terminals in the retina (Danbolt, 2001). In addition to that, EAATs also exist in endothelial cells (Campos et al., 2011b). All types of EAATs that are having similar mechanism act on clearing glutamate from the synaptic cleft (Magi et al., 2019).

Extracellular glutamate levels are strictly controlled *via* EAATs. EAATs that are found in astrocytes that bind and sequester the neurotransmitter for processing and recycling, promote the glutamate reuptake from synaptic junctions after neuronal excitation. As an alternative, glutamate can be transferred from the extracellular space *via* sodium-dependent transport on the antiluminal surface of brain capillary endothelial cells when the extracellular concentration is increased (Leibowitz et al., 2012). If the accumulation of glutamate concentration in the endothelial cells surpasses the plasma level, glutamate is transported from the luminal side into the bloodstream by facilitated diffusion. In this way, although there are adverse concentration gradients from central nervous system (CNS) to plasma, the endothelial regulation of CNS glutamate concentration can happen. After EAATs uptake glutamate from the synaptic cleft, it is converted in astrocytes to glutamine via glutamine synthetase or to α-ketoglutarate, which is involved in the tricarboxylic citric acid cycle (TCA) *via* glutamate dehydrogenase.

**FIGURE 3:**
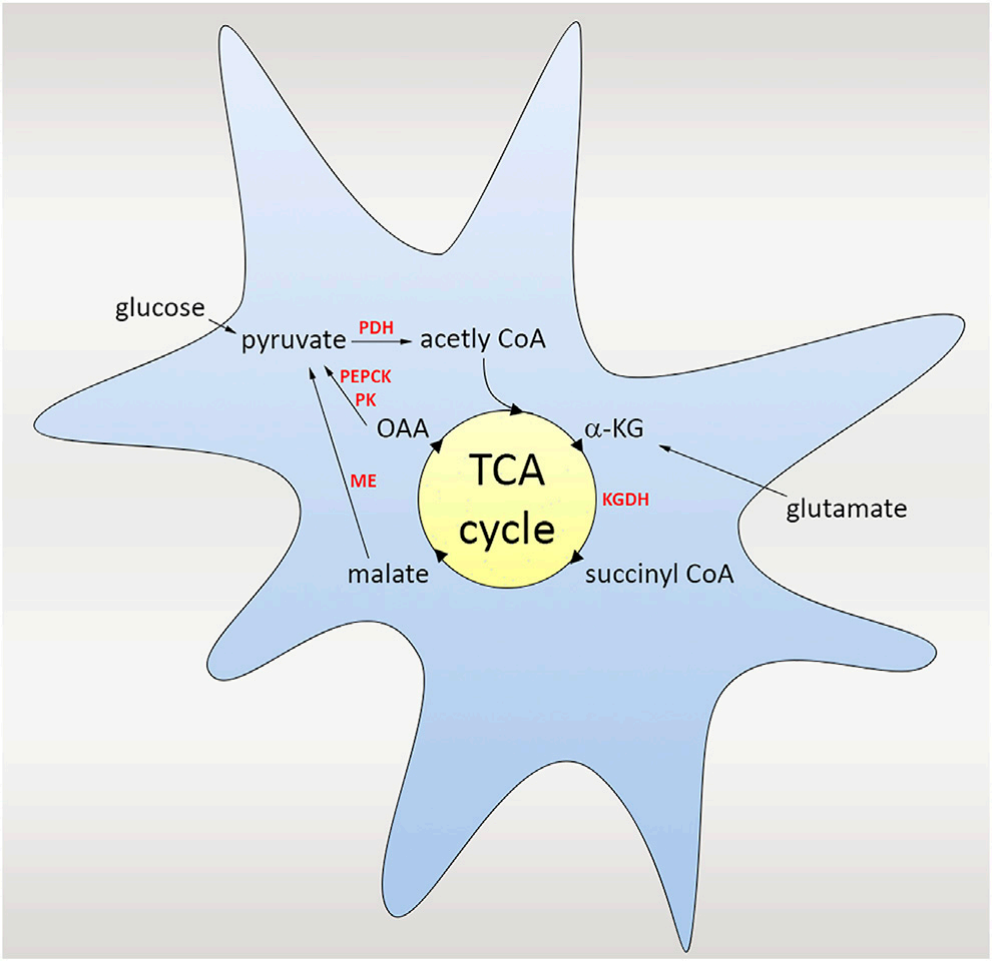
Scheme of the glutamate uptake and the TCA cycle. TCA cycle is a second stage of cellular respiration taking place in mitochondria, in which cells decompose organic molecules in the presence of oxygen and produce energy. The cycle starts with the catalyze of α-ketoglutarate to succinyl CoA *via* α-ketoglutarate dehydrogenase (KGDH), followed by several steps in the TCA cycle converting the carbon skeleton to malate, which is ultimately turned to pyruvate by malic enzyme (ME). As an alternative, pyruvate kinase (PK) and phosphoenolpyruvate carboxykinase (PEPCK) work and convert oxaloacetate (OAA) to pyruvate. Pyruvate dehydrogenase (PDH) also re-enters the TCA cycle, and the carbon skeleton derived from glutamate could be oxidized to CO_2._

TCA cycle, also called as Krebs cycle or citric acid cycle (CAC) is a second stage of cellular respiration, in which cells decompose organic molecules in the presence of oxygen and produce energy to be able to survive. This event takes place in mitochondria in almost all living organisms including most of the bacteria. The TCA cycle starts with the catalyze of α-ketoglutarate to succinyl CoA via α-ketoglutarate dehydrogenase (KGDH). Multiple steps in the tricarboxylic acid (TCA) cycle convert the carbon skeleton to malate, which is ultimately turned to pyruvate by malic enzyme (ME). As an alternative, pyruvate kinase (PK) and phosphoenolpyruvate carboxykinase (PEPCK) work together to convert oxaloacetate (OAA) to pyruvate. For the complete oxidation of glutamate, ME or PEPCK with PK must operate in the direction of pyruvate. Pyruvate dehydrogenase (PDH) re-enters the TCA cycle, and the carbon skeleton derived from glutamate can be entirely oxidized to CO_2_ in the TCA cycle through this pathway (Schousboe et al., 2014; Cooper and Jeitner, 2016) (Figure 3).

### Current Therapeutic Approaches in Ischemic Stroke

**FIGURE 4:**
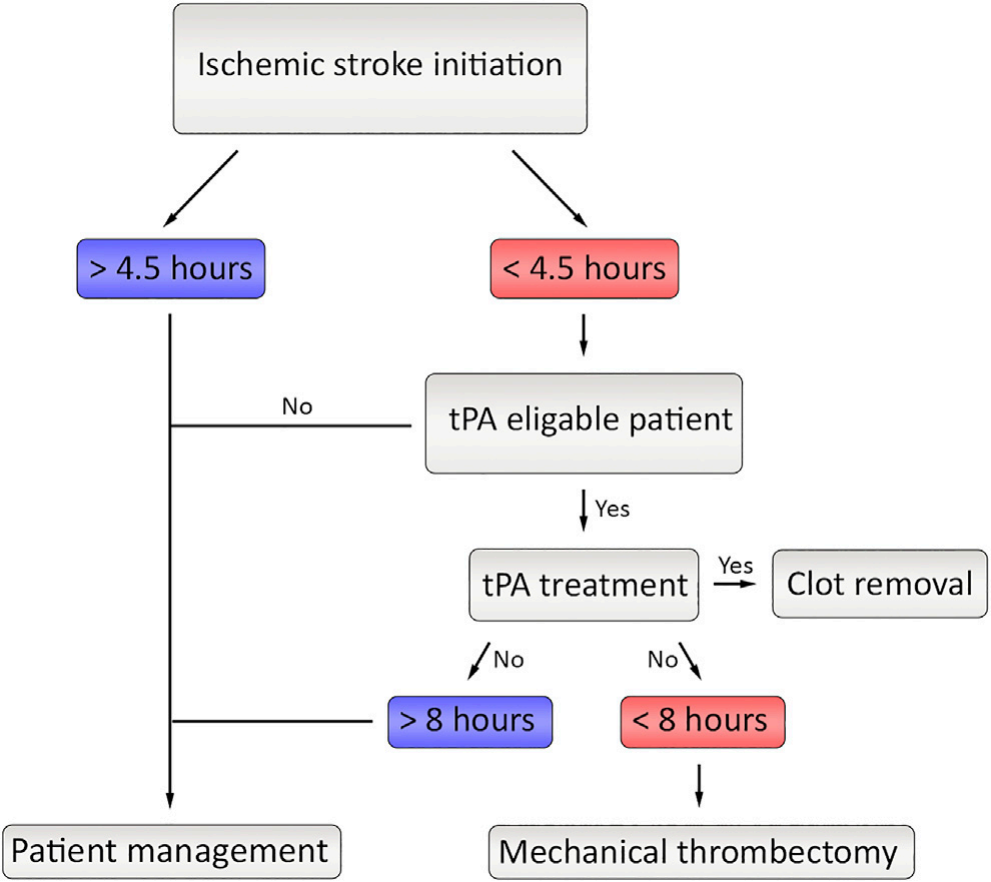
The current flow of therapeutic approaches in ischemic stroke. After initiation of the stroke, if the time limit is under 4.5 h and if tPA is eligible, the tPA treatment is applied. If the clot is still not fully removed and if the time did not exceed 8 h, Mechanical thrombectomy could be applied.

The treatment approach of stroke depends on the underlying cause of the disease because of the contradictory therapeutic requirements of hemorrhagic and ischemic types. While the hemorrhagic type requires immediate termination of bleeding, the ischemic type needs quick restoration of blood flow. The contrast between the motivations of treatment approaches makes the medical examination highly critical where misjudgment could worsen the existing damage on the brain. Once ischemia is identified, the medications aim the reperfusion of the affected area by either thrombolytic agents or mechanical thrombectomy (Dong et al., 2020) (Figure 4). Although there are several thrombolytic agents in the stage of clinical trials including alteplase, streptokinase and tenecteplase; only alteplase was approved by FDA. While streptokinase is eliminated because of the unacceptably high rates of hemorrhage, tenecteplase still requires further studies to verify benefits on ischemic stroke patients (Bhaskar et al., 2018).

### Alteplase (Tissue Plasminogen Activator, tPA)

By the day, tissue plasminogen activator (tPA or alteplase) is the only approved drug by the FDA and the golden standard of ischemic stroke treatment. tPA is a member of plasminogen activators that hydrolyze de arginine-valine peptide bond to activate plasminogen as plasmin (Lijnen and Collen, 1988). When given intravenously (IV), the tPA therapy can lyse the ischemia reasoning clot if it is performed within 3 h starting from the initiation of symptoms (National Institute of Neurological Disorders and Stroke rt-PA Stroke Study Group, 1995; Kwiatkowski et al., 1999; Bansal et al., 2013). If tPA treatment is applied directly to the affected area, therapy is generally accepted as successful up to the 4.5 h (Hacke et al., 2008). Although tPA therapy is the sharpest tool in the box, it is far from being perfect because thrombolytic therapy is still associated with bleeding that could be catastrophic (Goldstein et al., 2010). Therefore, there is a desperate and urgent need of new therapies in ischemic stroke.

### Mechanical Thrombectomy

Mechanical thrombectomy is a minimally invasive procedure that includes removal of the clot from the artery of the patient by using a microcatheter. The size of the clot can differ depending on the patient, and if it too big or tPA treatment time is surpassed, MT surgery is the only treatment that could be applied if the onset is before 8 h (Jovin et al., 2015). It has been observed that MT treatment supplied two times more survival rate and less disability in patients diagnosed with ischemic stroke (Smith et al., 2008). MT and pre-treatment with intravenous tPA treatment could be another therapeutic option. However, this therapy is also disadvantageous since handling the clot within the vessel could be risky in terms of mechanical injuries (Bhaskar et al., 2018).

### Peritoneal Dialysis

There is another strategy called as peritoneal dialysis have been developed in order to decrease blood glutamate levels by directing glutamate from brain to blood. The transient increase in glutamate is aimed to decrease via this dialysis method simply by exchanging fluids and dissolved substances between the blood and the dialysate across the peritoneum (Godino et al., 2013). It has been also demonstrated that this strategy significantly decrease glutamate levels in patients (Rogachev et al., 2013). This novel therapy can be an effective strategy for stroke patients, however it is not commonly used as a stroke therapy yet and clinical trials are needed to see the efficiancy in ischemic stroke patients.

### Stroke Prevention

Despite the fact that prevention of stroke is not a treatment itself, it is commonly used and quite effective therapeutic intervention method against stroke. Anticoagulants, antiplatelet therapy, statin treatment, drugs that are regulating blood pressure are currently used stroke prevention methods, and it has been foreseen that up to 80% of recurrent stroke cases can be averted by the usage of these methods. Therefore, mass stroke prevention strategy could be a very useful complementary strategy to decrease the increasing trend of global burden of stroke as well as subsequent disability (Sherzai and Elkind, 2015).

### Neuroprotective Therapies

Significant pharmacological strategies to this neurotoxic event have been improved by researchers thanks to the knowledge of the molecular mechanisms contained in glutamate excitotoxicity after cerebral ischemia. Initially, NMDA receptors (NMDAR) antagonism was investigated as a main focus; since NMDAR is a significant gateway for the numerous other downstream effects of glutamate excitotoxicity. Therefore, it ensured a reasonable target for drug design. Throughout this period, the knowledge of the structure and function of these receptors also increased owing to advances in protein biochemistry and small molecule design (Ogden and Traynelis, 2011). Various classes of NMDAR antagonists that have different sites of action, were improved that are the competitive NMDAR antagonist affecting for glutamate or glycine binding sites, noncompetitive allosteric inhibitors acting on other extracellular sites, and NMDAR channel blockers that influence sites in the receptor channel pore. Although there are promising results in animal research, antagonist drugs like selfotel, gavestinel, and traxoprodil, have not dramatically succeeded in randomized and controlled clinical trials in humans. The failure of these NMDAR-targeting therapies has been explained with many different reasons. Some of them show important dose-limiting side effects and lack adequate brain penetration (Kalia et al., 2008; Grupke et al., 2015; Jia et al., 2015). There are also some unfavorable process profiles such as hallucinations, agitations, catatonia, peripheral sensory loss, nausea, and elevation in blood pressure (Muir, 2006). Furthermore, it is thought that glutamate excitotoxicity leads to harm in a narrow time frame during which the neurotransmitter performs its normal function in the condition of acute processes like stroke or traumatic brain injuries. Thus, unfavorable side effects due to the prolonged receptor blockade and less therapeutic efficacy may occur by using agents acting on NMDAR, which is a fundamental receptor of glutamate. Although there were initial failures, research about NMDAR antagonism has been conducted. Recent experiments focusing on the control of the upstream glutamate concentration and downstream protein signals have begun to expand beyond the NMDAR over the past 2 decades. Novel theraphy approaches related to NMDA receptors including death-signaling pathways have been developed. These downstream pathyways of the receptor are inhibited by using inhibitors without blocking NMDARs such as interfering peptides and pharmacological inhibitors. These agents particularly uncouple the neuronal death signaling from NMDARs and they don’t change neither functional nor survival of the receptors. In contrast to the receptor antagonist therapies, this approach may have less side effects and also has a potential to supply a longer therapeutic effect for stroke (Wu and Tymianski, 2018). Currently, there is increasing interest in blood glutamate scavengers and other agents targeting downstream sites of the receptor to improve effective drug modalities (Ikonomidou and Turski, 2002; Korczyn et al., 2015; Castillo et al., 2016).

## Glutamate Scavenging in Ischemic Stroke

### Definition and the Mechanism of Scavenging

**FIGURE 5:**
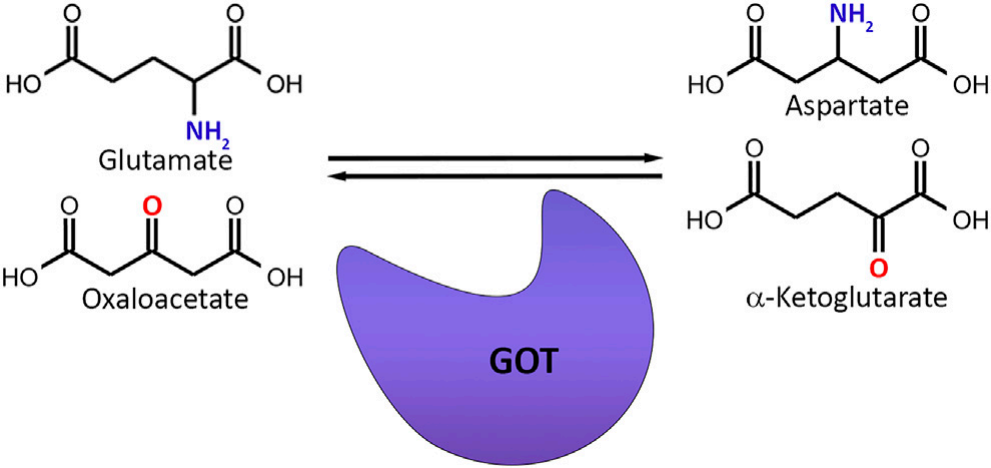
Basic mechanism of glutamate scavenging. Glutamate-oxaloacetate Transaminase (GOT) transfers the amino group from glutamate to oxaloacetate while replacing the amino group of glutamate with a carbonyl group and converts glutamate and oxaloacetate to aspartate and α-ketoglutarate.

The word scavenger means the elimination of unwanted substances in order to keep the biological environment stable. Since glutamate is the major element to control in the CNS, regulating glutamate levels by a scavenging mechanism would be a very smart way to preserve homeostasis. The basis of systemic glutamate scavenging is based on a simple reaction, in which a blood-resident enzyme glutamate-oxaloacetate transaminase (GOT), also referred as Aspartate Aminotransferase (AST) converts glutamate into α-ketoglutarate and aspartate in existence of oxaloacetate (Campos et al., 2012) (Figure 5). The GOT enzyme-based reaction is dependent to the pyridoxal-5-phosphate (P5P) cofactor, which is the active type of vitamin B6. It binds covalently to an active-site lysine in a reversible manner and help catalyze the reaction (Westerhuis and Hafkenscheid, 1983; Tutor-Crespo et al., 2004).

The main advantage of glutamate scavengers is that they are doing their job in the blood. Furthermore, this mechanism acts locally in the brain regions that have a high glutamate concentration, whereas other brain regions that have normal glutamate levels have been unaffected, which eliminates secondary effects by decreasing cumulative glutamate levels in the brain. The glutamate concentration in blood plasma is measured as 40–60 μM in healthy adults (Leibowitz et al., 2012; Bai et al., 2017), whereas glutamate levels in cerebrospinal fluid (CSF) or brain intercellular fluids is between 1 and 10 μM (Hawkins, 2009; Teichberg et al., 2009) revealing that the glutamate concentration is much higher in the blood than in the CSF. Due to that difference, there is intraparenchymal-blood glutamate concentration gradient occurring between blood and brain, and it is mainly dependent to the integrity of the BBB as well as endothelial glutamate transporters (EAATs) (Hawkins, 2009; Cohen-Kashi-Malina et al., 2012; Bai and Zhou, 2017). When stroke happens, glutamate concentration in the brain goes up to 10 times above normal that which leads to a higher glutamate concentration is higher in the brain side than the blood (Campos et al., 2012). Another advantage of glutamate scavengers is that the systemic glutamate scavenging process is self-controlled, and automatically stops if that imbalance between brain and blood glutamate levels is reverted to the normal. One of the most important features of this method is, these scavengers do not give any harm, meaning that if there is a stroke risk observed, these agents can be given for prevention of the disease safely. Compared to previously used NMDA receptor antagonists that are not successfully working as well as many side effects since their interference with regular receptor signaling, systemic glutamate scavenging may be the smartest way for future glutamate excitotoxicity treatment (Boyko et al., 2014).

## Current Glutamate Scavengers

### Glutamate-Oxaloacetate Transaminase and Glutamate-Pyruvate Transaminase

Based on the systemic glutamate scavenging method, it was first demonstrated that, upon glutamate increase in CSF, GOT and glutamate-pyruvate transaminase (GPT) lead to a significant reduction of glutamate in blood, further diminishing the extracellular glutamate amount in brain (Gottlieb et al., 2003). GOT and GPT, together with the addition of their co-substrates pyruvate and oxaloacetate, transform glutamate to α-ketoglutarate and aspartate. This was the starting point for the potential systemic glutamate scavenging method for the eradication of the redundant glutamate in the brain ventricles as well as CSF. Afterwards, there has been a focus on glutamate converting transaminases as a possible therapeutic approach. It has been shown that peripheral administration of GOT and GPT with and without oxaloacetate and pyruvate give rise to a decrease in blood glutamate levels (Zlotnik et al., 2007; Boyko et al., 2012a; Boyko et al., 2012b).

One of the interesting findings has come recently that by doing both transcriptome screening and qPCR, GOT levels are found to be increased in MCAO rat ischemic stroke model (Rink et al., 2010). This study is followed by a unique *in vitro* study and by using neuronal HT4 cells transfected with GOT siRNA and verifying diminished GOT mRNA level and GOT activity, neuroprotective effect of GOT under hypoglycemic and high extracellular glutamate conditions has been discovered (Rink et al., 2011). Another study elucidated that patients with big infarction have higher glutamate and lower GOT levels in blood due to the capacity of the to metabolize blood glutamate, which makes GOT a potential biomarker showing the severity of the stroke (Campos et al., 2011c). There has also been a comparison of GOT and GPT in ischemic stroke patients, highlighting that the effect of GOT levels are more significantly showing a better outcome than GPT levels (Campos et al., 2011b). Yet, *in vitro* mechanism of GOT still needs to be understood.

Very recently, neuroprotective outcome of a bioconjugate human recombinant glutamate oxaloacetate transaminase (hrGOT) has been studied and it has been observed that a single administration of hrGOT leads to a drastic decrease of glutamate in bloodstream up to a week. Neuroprotective effect of hrGOT has also been confirmed by decreased infarct volume and improved sensorimotor functions in ischemic rats. Interestingly, the innovative form, the blood brain barrier targeted hrGOT did not show a significant increase in terms of efficiency, stating that glutamate scavenging activity in blood is an effective and powerful technique for stroke neuroprotection (Zaghmi et al., 2020).

### Oxaloacetate

The neuroprotective role of oxaloacetate in ischemia is shown in rats by photothrombotic lesion and revealed that it reduces both blood and brain glutamate levels (Nagy et al., 2009). Interestingly, oxaloacetate injection in complete forebrain ischemia (2VO) model does also provide reestablishment of synaptic plasticity measured by LTP recordings in the CA1 region of hippocampus (Marosi et al., 2009). Further studies are done to be able to show the neuroprotective effect of oxaloacetate on diminishing blood as well as brain glutamate, by transient occlusion of the middle cerebral artery (MCAO) ischemic model, it has been observed that intravenous oxaloacetate administration leads to a significant drop of glutamate levels both in blood and brain. Moreover, it is confirmed by magnetic resonance spectroscopy (MRS) of the brain clearly showing the neuroprotective outcome of oxaloacetate (Campos et al., 2011a). It has also been demonstrated that glutamate levels in brain extracellular fluid (ECF) can be cleared by oxaloacetate (Teichberg et al., 2009). In another study in which maleate, a glutamate-oxaloacetate transaminase-blocker used next to oxaloacetate elucidates that the neuroprotective activity is reversed, proving the systemic glutamate scavenging activity of oxaloacetate (Zlotnik et al., 2009). Neuroprotective effect of oxaloacetate is also confirmed by electrophysiological methods measuring somatosensory evoked responses upon MCAO, and histological methods (Campos et al., 2012; Knapp et al., 2015; Castillo et al., 2016).

### Pyruvate

**FIGURE 6:**
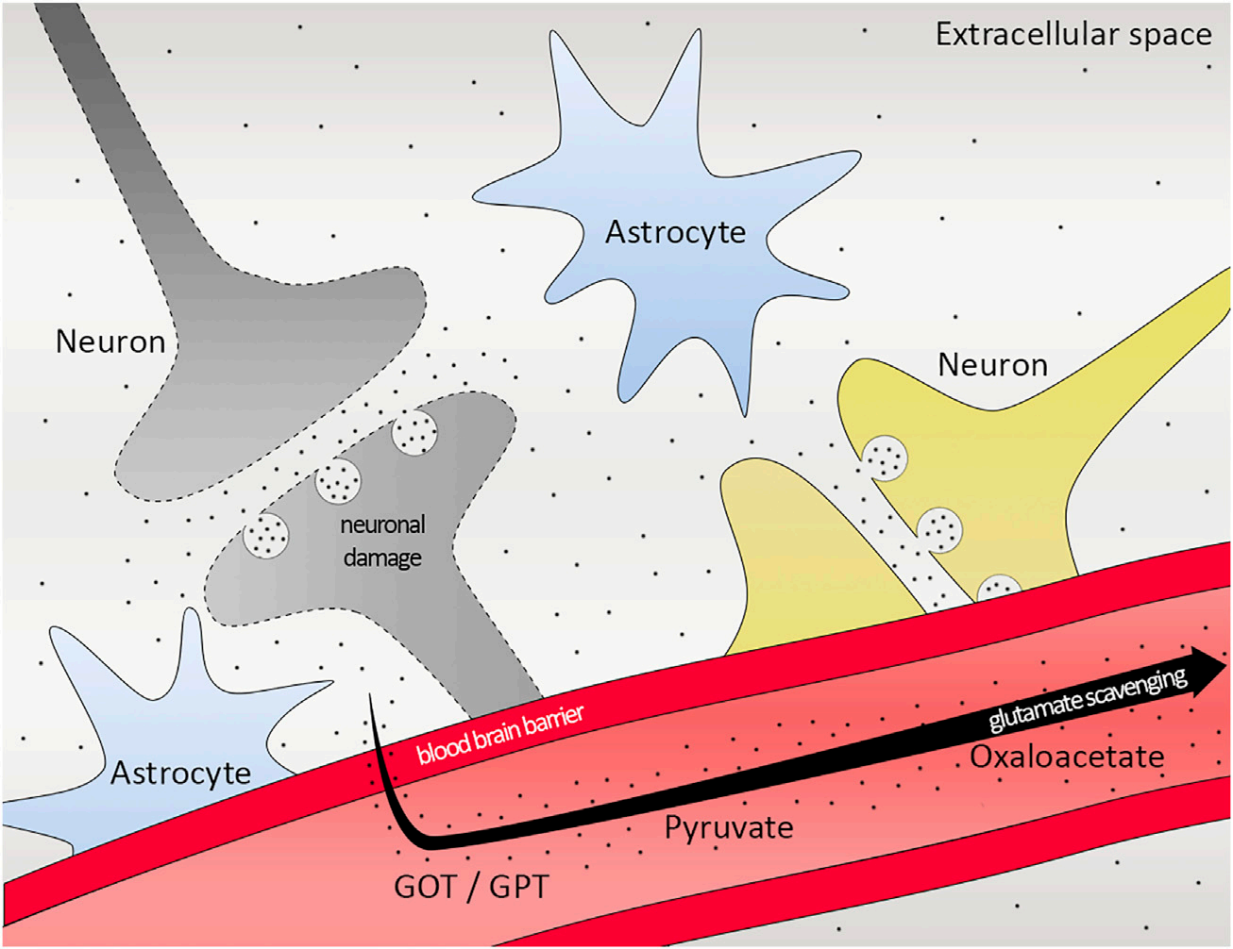
Scheme of glutamate scavenging. Working mechanism of GOT/GPT, pyruvate and oxaloacetate in the brain. Black dots representing glutamate. GOT, Glutamate-oxaloacetate transaminase; GPT, Glutamate-pyruvate transaminase.

The neuroprotective feature of pyruvate in ischemic stroke has been discovered 2 decades ago (Lee et al., 2001). It is followed by more studies showing the protective effect (Yi et al., 2007), as well as by another pyruvate derivative, ethyl pyruvate has also a role in neuroprotective outcome (Kim et al., 2005). However, the glutamate scavenging role of pyruvate is elucidated later by a rat model with closed head injury (Zlotnik et al., 2008). More studies have been done stating that glutamate–pyruvate transaminase together with pyruvate has a protective effect on ischemic rats after head trauma (Boyko et al., 2011), all in all emphasizing the therapeutic potential of glutamate grabber pyruvate in ischemic stroke (Figure 6).

## Conclusion and Future Prospects

Ischemic stroke, one of the major reasons of death and disability worldwide, occurs because of the blockage of blood supply to the brain. Glucose and oxygen are limited by the decreased blood flow, causing insufficient energy supply in neurons. Neurons can no longer sustain their transmembrane gradient, leading to depolarization, which induces excitotoxicity *via* releasing neurotransmitters, especially glutamate. Glutamate excitotoxicity increases the production of harmful substances such as ROS that leads to endothelial apoptosis and blood-brain barrier damage and enhances the cerebral injury. Administration of tPA is still one and only therapy for ischemic stroke and the time frame is really short. Recent studies focusing on the glutamate excitotoxicity generates very promising data and scientists are improving novel therapeutic agents targeting different steps in the pathway. In this review, we sought to describe major strategies that are in development, namely systemic glutamate scavenging, which directly decreases extracellular glutamate in the CNS. In particular, systemic glutamate scavenging has received much attention as a new approach to prevent excitotoxicity. Moreover, they are extremely useful due to the fact that there are no side effects, which allow us to use them immediately if suspected and making therapy available for everybody. Since these different methods target to influence the glutamate-mediated damage at different levels, it may be interesting to explore the use of drug combinations to seek synergies in the future. Those trials will allow us to elucidate the application of this new therapeutic strategy. Last but not least, it is also crucial to understand the molecular mechanism behind the glutamate scavengers as well as permeability on blood brain barrier to be able to broaden the window of the current and future therapeutic options in ischemic stroke.

## References

[B1] Appireddy R. M. Demchuk A. M. Goyal M. Menon B. K. Eesa M. Choi P. (2015). Endovascular Therapy for Ischemic Stroke. J. Clin. Neurol. 11 (1), 1–8. 10.3988/jcn.2015.11.1.1 25628731 PMC4302170

[B2] Atoji Y. Sarkar S. (2019). Localization of AMPA, Kainate, and NMDA Receptor mRNAs in the pigeon Cerebellum. J. Chem. Neuroanat. 98, 71–79. 10.1016/j.jchemneu.2019.04.004 30978490

[B3] Bai W. Zhou Y. G. (2017). Homeostasis of the Intraparenchymal-Blood Glutamate Concentration Gradient: Maintenance, Imbalance, and Regulation. Front. Mol. Neurosci. 10, 400. 10.3389/fnmol.2017.00400 29259540 PMC5723322

[B4] Bai W. Zhu W. L. Ning Y. L. Li P. Zhao Y. Yang N. (2017). Dramatic Increases in Blood Glutamate Concentrations Are Closely Related to Traumatic Brain Injury-Induced Acute Lung Injury. Sci. Rep. 7 (1), 5380. 10.1038/s41598-017-05574-9 28710466 PMC5511144

[B5] Bansal S. Sangha K. S. Khatri P. (2013). Drug Treatment of Acute Ischemic Stroke. Am. J. Cardiovasc. Drugs 13 (1), 57–69. 10.1007/s40256-013-0007-6 23381911 PMC3840541

[B6] Belov Kirdajova D. Kriska J. Tureckova J. Anderova M. (2020). Ischemia-Triggered Glutamate Excitotoxicity from the Perspective of Glial Cells. Front Cel Neurosci 14, 51. 10.3389/fncel.2020.00051 PMC7098326 32265656

[B7] Bhaskar S. Stanwell P. Cordato D. Attia J. Levi C. (2018). Reperfusion Therapy in Acute Ischemic Stroke: Dawn of a new era? BMC Neurol. 18 (1), 8. 10.1186/s12883-017-1007-y 29338750 PMC5771207

[B8] Boyko M. Gruenbaum S. E. Gruenbaum B. F. Shapira Y. Zlotnik A. (2014). Brain to Blood Glutamate Scavenging as a Novel Therapeutic Modality: a Review. J. Neural Transm. (Vienna). 971–979. 121. 10.1007/s00702-014-1181-7 24623040 PMC4382077

[B9] Boyko M. Melamed I. Gruenbaum B. F. Gruenbaum S. E. Ohayon S. Leibowitz A. (2012a). The Effect of Blood Glutamate Scavengers Oxaloacetate and Pyruvate on Neurological Outcome in a Rat Model of Subarachnoid Hemorrhage. Neurotherapeutics 9 (3), 649–657. 10.1007/s13311-012-0129-6 22711471 PMC3441925

[B10] Boyko M. Stepensky D. Gruenbaum B. F. Gruenbaum S. E. Melamed I. Ohayon S. (2012b). Pharmacokinetics of Glutamate-Oxaloacetate Transaminase and Glutamate-Pyruvate Transaminase and Their Blood Glutamate-Lowering Activity in Naïve Rats. Neurochem. Res. 37 (10), 2198–2205. 10.1007/s11064-012-0843-9 22846966

[B11] Boyko M. Zlotnik A. Gruenbaum B. F. Gruenbaum S. E. Ohayon S. Kuts R. (2011). Pyruvate's Blood Glutamate Scavenging Activity Contributes to the Spectrum of its Neuroprotective Mechanisms in a Rat Model of Stroke. Eur. J. Neurosci. 34 (9), 1432–1441. 10.1111/j.1460-9568.2011.07864.x 21936878

[B12] Campos F. Rodríguez-Yáñez M. Castellanos M. Arias S. Pérez-Mato M. Sobrino T. (2011a). Blood Levels of Glutamate Oxaloacetate Transaminase Are More Strongly Associated with Good Outcome in Acute Ischaemic Stroke Than Glutamate Pyruvate Transaminase Levels. Clin. Sci. (Lond) 121 (1), 11–17. 10.1042/CS20100427 21265738

[B13] Campos F. Sobrino T. Ramos-Cabrer P. Argibay B. Agulla J. Pérez-Mato M. (2011b). Neuroprotection by Glutamate Oxaloacetate Transaminase in Ischemic Stroke: an Experimental Study. J. Cereb. Blood Flow Metab. 31 (6), 1378–1386. 10.1038/jcbfm.2011.3 21266983 PMC3130324

[B14] Campos F. Sobrino T. Ramos-Cabrer P. Castellanos M. Blanco M. Rodríguez-Yáñez M. (2011c). High Blood Glutamate Oxaloacetate Transaminase Levels Are Associated with Good Functional Outcome in Acute Ischemic Stroke. J. Cereb. Blood Flow Metab. 31 (6), 1387–1393. 10.1038/jcbfm.2011.4 21266984 PMC3130328

[B15] Campos F. Sobrino T. Ramos-Cabrer P. Castillo J. (2012). Oxaloacetate: a Novel Neuroprotective for Acute Ischemic Stroke. Int. J. Biochem. Cel Biol 44 (2), 262–265. 10.1016/j.biocel.2011.11.003 22085530

[B16] Castillo J. Loza M. I. Mirelman D. Brea J. Blanco M. Sobrino T. (2016). A Novel Mechanism of Neuroprotection: Blood Glutamate Grabber. J. Cereb. Blood Flow Metab. 36 (2), 292–301. 10.1177/0271678X15606721 26661174 PMC4759671

[B17] Cohen-Kashi-Malina K. Cooper I. Teichberg V. I. (2012). Mechanisms of Glutamate Efflux at the Blood-Brain Barrier: Involvement of Glial Cells. J. Cereb. Blood Flow Metab. 32 (1), 177–189. 10.1038/jcbfm.2011.121 21915136 PMC3323299

[B18] Cooper A. J. Jeitner T. M. (2016). Central Role of Glutamate Metabolism in the Maintenance of Nitrogen Homeostasis in Normal and Hyperammonemic Brain. Biomolecules 6 (2), 16. 10.3390/biom6020016 PMC4919911 27023624

[B19] Danbolt N. C. (2001). Glutamate Uptake. Prog. Neurobiol. 65 (1), 1–105. 10.1016/s0301-0082(00)00067-8 11369436

[B20] Danbolt N. C. Pines G. Kanner B. I. (1990). Purification and Reconstitution of the Sodium- and Potassium-Coupled Glutamate Transport Glycoprotein from Rat Brain. Biochemistry 29 (28), 6734–6740. 10.1021/bi00480a025 1697765

[B21] Dong X. Gao J. Su Y. Wang Z. (2020). Nanomedicine for Ischemic Stroke. Int. J. Mol. Sci. 21 (20), 7600. 10.3390/ijms21207600 PMC7590220 33066616

[B22] Doyle K. P. Simon R. P. Stenzel-Poore M. P. (2008). Mechanisms of Ischemic Brain Damage. Neuropharmacology 55 (3), 310–318. 10.1016/j.neuropharm.2008.01.005 18308346 PMC2603601

[B23] Girling K. D. Demers M. J. Laine J. Zhang S. Wang Y. T. Graham R. K. (2018). Activation of Caspase-6 and Cleavage of Caspase-6 Substrates Is an Early Event in NMDA Receptor-Mediated Excitotoxicity. J. Neurosci. Res. 96 (3), 391–406. 10.1002/jnr.24153 29193273

[B24] Global GBD 2016 Stroke Collaborators (2019). Global, Regional, and National burden of Stroke, 1990-2016: a Systematic Analysis for the Global Burden of Disease Study 2016. Lancet Neurol. 18 (5), 439–458. 10.1016/S1474-4422(19)30034-1 30871944 PMC6494974

[B25] Godino Mdel. C. Romera V. G. Sánchez-Tomero J. A. Pacheco J. Canals S. Lerma J. (2013). Amelioration of Ischemic Brain Damage by Peritoneal Dialysis. J. Clin. Invest. 123 (10), 4359–4363. 10.1172/JCI67284 23999426 PMC3784528

[B26] Goldstein J. N. Marrero M. Masrur S. Pervez M. Barrocas A. M. Abdullah A. (2010). Management of Thrombolysis-Associated Symptomatic Intracerebral Hemorrhage. Arch. Neurol. 67 (8), 965–969. 10.1001/archneurol.2010.175 20697046 PMC3690951

[B27] Gottlieb M. Wang Y. Teichberg V. I. (2003). Blood-mediated Scavenging of Cerebrospinal Fluid Glutamate. J. Neurochem. 87 (1), 119–126. 10.1046/j.1471-4159.2003.01972.x 12969259

[B28] Grewer C. Rauen T. (2005). Electrogenic Glutamate Transporters in the CNS: Molecular Mechanism, Pre-steady-state Kinetics, and Their Impact on Synaptic Signaling. J. Membr. Biol. 203 (1), 1–20. 10.1007/s00232-004-0731-6 15834685 PMC2389879

[B29] Grupke S. Hall J. Dobbs M. Bix G. J. Fraser J. F. (2015). Understanding History, and Not Repeating it. Neuroprotection for Acute Ischemic Stroke: from Review to Preview. Clin. Neurol. Neurosurg. 129, 1–9. 10.1016/j.clineuro.2014.11.013 25497127

[B30] Gupta K. Hardingham G. E. Chandran S. (2013). NMDA Receptor-dependent Glutamate Excitotoxicity in Human Embryonic Stem Cell-Derived Neurons. Neurosci. Lett. 543, 95–100. 10.1016/j.neulet.2013.03.010 23518152 PMC3725411

[B31] Hacke W. Kaste M. Bluhmki E. Brozman M. Dávalos A. Guidetti D. (2008). Thrombolysis with Alteplase 3 to 4.5 Hours after Acute Ischemic Stroke. N. Engl. J. Med. 359 (13), 1317–1329. 10.1056/NEJMoa0804656 18815396

[B32] Hawkins R. A. (2009). The Blood-Brain Barrier and Glutamate. Am. J. Clin. Nutr. 90 (3), 867S–874S. 10.3945/ajcn.2009.27462BB 19571220 PMC3136011

[B33] Heiss W. D. Rosner G. (1983). Functional Recovery of Cortical Neurons as Related to Degree and Duration of Ischemia. Ann. Neurol. 14 (3), 294–301. 10.1002/ana.410140307 6314871

[B34] Ikonomidou C. Turski L. (2002). Why Did NMDA Receptor Antagonists Fail Clinical Trials for Stroke and Traumatic Brain Injury? Lancet Neurol. 1 (6), 383–386. 10.1016/s1474-4422(02)00164-3 12849400

[B35] Jia M. Njapo S. A. Rastogi V. Hedna V. S. (2015). Taming Glutamate Excitotoxicity: Strategic Pathway Modulation for Neuroprotection. CNS drugs 29 (2), 153–162. 10.1007/s40263-015-0225-3 25633850

[B36] Jovin T. G. Chamorro A. Cobo E. de Miquel M. A. Molina C. A. Rovira A. (2015). Thrombectomy within 8 hours after Symptom Onset in Ischemic Stroke. N. Engl. J. Med. 372 (24), 2296–2306. 10.1056/NEJMoa1503780 25882510

[B37] Kalia L. V. Kalia S. K. Salter M. W. (2008). NMDA Receptors in Clinical Neurology: Excitatory Times Ahead. Lancet Neurol. 7 (8), 742–755. 10.1016/S1474-4422(08)70165-0 18635022 PMC3589564

[B38] Kim J. B. Yu Y. M. Kim S. W. Lee J. K. (2005). Anti-inflammatory Mechanism Is Involved in Ethyl Pyruvate-Mediated Efficacious Neuroprotection in the Postischemic Brain. Brain Res. 1060 (1-2), 188–192. 10.1016/j.brainres.2005.08.029 16226231

[B39] Knapp L. Gellért L. Kocsis K. Kis Z. Farkas T. Vécsei L. (2015). Neuroprotective Effect of Oxaloacetate in a Focal Brain Ischemic Model in the Rat. Cell Mol Neurobiol 35 (1), 17–22. 10.1007/s10571-014-0064-7 24807461 PMC11486343

[B40] Korczyn A. D. Brainin M. Guekht. A. (2015). Neuroprotection in Ischemic Stroke: what Does the Future Hold? Expert Rev. Neurother 15 (3), 227–229. 10.1586/14737175.2015.1014806 25708307

[B41] Kurth T. Heuschmann P. U. Walker A. M. Berger K. (2007). Mortality of Stroke Patients Treated with Thrombolysis: Analysis of Nationwide Inpatient Sample. Neurology 68 (9), 710–711. 10.1212/01.wnl.0000258816.02021.b9 17325289

[B42] Kwiatkowski T. G. Libman R. B. Frankel M. Tilley B. C. Morgenstern L. B. Lu M. (1999). Effects of Tissue Plasminogen Activator for Acute Ischemic Stroke at One Year. National Institute of Neurological Disorders and Stroke Recombinant Tissue Plasminogen Activator Stroke Study Group. N. Engl. J. Med. 340 (23), 1781–1787. 10.1056/NEJM199906103402302 10362821

[B43] Lee J. Y. Kim Y. H. Koh J. Y. (2001). Protection by Pyruvate against Transient Forebrain Ischemia in Rats. J. Neurosci. 21 (20), RC171. 10.1523/jneurosci.21-20-j0002.2001 11588201 PMC6763857

[B44] Leibowitz A. Boyko M. Shapira Y. Zlotnik A. (2012). Blood Glutamate Scavenging: Insight into Neuroprotection. Int. J. Mol. Sci. 13 (8), 10041–10066. 10.3390/ijms130810041 22949847 PMC3431845

[B45] Lijnen H. R. Collen D. (1988). Mechanisms of Plasminogen Activation by Mammalian Plasminogen Activators. Enzyme 40 (2-3), 90–96. 10.1159/000469150 3139404

[B46] Luoma J. I. Kelley B. G. Mermelstein P. G. (2011). Progesterone Inhibition of Voltage-Gated Calcium Channels Is a Potential Neuroprotective Mechanism against Excitotoxicity. Steroids 76 (9), 845–855. 10.1016/j.steroids.2011.02.013 21371490 PMC3129396

[B47] Magi S. Piccirillo S. Amoroso S. Lariccia V. (2019). Excitatory Amino Acid Transporters (EAATs): Glutamate Transport and beyond. Int. J. Mol. Sci. 20 (22), 5674. 10.3390/ijms20225674 PMC6888595 31766111

[B48] Marosi M. Fuzik J. Nagy D. Rákos G. Kis Z. Vécsei L. (2009). Oxaloacetate Restores the Long-Term Potentiation Impaired in Rat hippocampus CA1 Region by 2-vessel Occlusion. Eur. J. Pharmacol. 604 (1-3), 51–57. 10.1016/j.ejphar.2008.12.022 19135048

[B49] Matei N. Camara J. Zhang J. H. (2020). The Next Step in the Treatment of Stroke. Front. Neurol. 11, 582605. 10.3389/fneur.2020.582605 33551950 PMC7862333

[B50] Mennerick S. Dhond R. P. Benz A. Xu W. Rothstein J. D. Danbolt N. C. (1998). Neuronal Expression of the Glutamate Transporter GLT-1 in Hippocampal Microcultures. J. Neurosci. 18 (12), 4490–4499. 10.1523/jneurosci.18-12-04490.1998 9614226 PMC6792702

[B51] Muir K. W. (2006). Glutamate-based Therapeutic Approaches: Clinical Trials with NMDA Antagonists. Curr. Opin. Pharmacol. 6 (1), 53–60. 10.1016/j.coph.2005.12.002 16359918

[B52] Nagy D. Marosi M. Kis Z. Farkas T. Rakos G. Vecsei L. (2009). Oxaloacetate Decreases the Infarct Size and Attenuates the Reduction in Evoked Responses after Photothrombotic Focal Ischemia in the Rat Cortex. Cel Mol Neurobiol 29 (6-7), 827–835. 10.1007/s10571-009-9364-8 PMC11506091 19259807

[B53] National Institute of Neurological Disorders and Stroke rt-PA Stroke Study Group (1995). Tissue Plasminogen Activator for Acute Ischemic Stroke. N. Engl. J. Med. 333 (24), 1581–1587. 10.1056/NEJM199512143332401 7477192

[B54] Ogden K. K. Traynelis S. F. (2011). New Advances in NMDA Receptor Pharmacology. Trends Pharmacol. Sci. 32 (12), 726–733. 10.1016/j.tips.2011.08.003 21996280 PMC3223280

[B55] Papazian I. Kyrargyri V. Evangelidou M. Voulgari-Kokota A. Probert L. (2018). Mesenchymal Stem Cell Protection of Neurons against Glutamate Excitotoxicity Involves Reduction of NMDA-Triggered Calcium Responses and Surface GluR1, and Is Partly Mediated by TNF. Int. J. Mol. Sci. 19 (3), 651. 10.3390/ijms19030651 PMC5877512 29495345

[B56] Pines G. Danbolt N. C. Bjørås M. Zhang Y. Bendahan A. Eide L. (1992). Cloning and Expression of a Rat Brain L-Glutamate Transporter. Nature 360 (6403), 464–467. 10.1038/360464a0 1448170

[B57] Rink C. Gnyawali S. Peterson L. Khanna S. (2011). Oxygen-inducible Glutamate Oxaloacetate Transaminase as Protective Switch Transforming Neurotoxic Glutamate to Metabolic Fuel during Acute Ischemic Stroke. Antioxid. Redox Signal. 14 (10), 1777–1785. 10.1089/ars.2011.3930 21361730 PMC3078502

[B58] Rink C. Roy S. Khan M. Ananth P. Kuppusamy P. Sen C. K. (2010). Oxygen-sensitive Outcomes and Gene Expression in Acute Ischemic Stroke. J. Cereb. Blood Flow Metab. 30 (7), 1275–1287. 10.1038/jcbfm.2010.7 20145654 PMC2913550

[B59] Rogachev B. Tsesis S. Gruenbaum B. F. Gruenbaum S. E. Boyko M. Klein M. (2013). The Effects of Peritoneal Dialysis on Blood Glutamate Levels: Implementation for Neuroprotection. J. Neurosurg. Anesthesiol 25 (3), 262–266. 10.1097/ANA.0b013e318283f86a 23752045

[B60] Rossi D. J. Brady J. D. Mohr C. (2007). Astrocyte Metabolism and Signaling during Brain Ischemia. Nat. Neurosci. 10 (11), 1377–1386. 10.1038/nn2004 17965658 PMC8906499

[B61] Rossi D. J. Oshima T. Attwell D. (2000). Glutamate Release in Severe Brain Ischaemia Is Mainly by Reversed Uptake. Nature 403 (6767), 316–321. 10.1038/35002090 10659851

[B62] Rothstein J. D. Martin L. Levey A. I. Dykes-Hoberg M. Jin L. Wu D. (1994). Localization of Neuronal and Glial Glutamate Transporters. Neuron 13 (3), 713–725. 10.1016/0896-6273(94)90038-8 7917301

[B63] Sattler R. Xiong Z. Lu W. Y. Hafner M. MacDonald J. F. Tymianski M. (1999). Specific Coupling of NMDA Receptor Activation to Nitric Oxide Neurotoxicity by PSD-95 Protein. Science 284 (5421), 1845–1848. 10.1126/science.284.5421.1845 10364559

[B64] Schousboe A. Scafidi S. Bak L. K. Waagepetersen H. S. McKenna M. C. (2014). Glutamate Metabolism in the Brain Focusing on Astrocytes. Adv. Neurobiol. 11, 13–30. 10.1007/978-3-319-08894-5_2 25236722 PMC4667713

[B65] Sherzai A. Z. Elkind M. S. (2015). Advances in Stroke Prevention. Ann. N. Y Acad. Sci. 1338, 1–15. 10.1111/nyas.12723 25779474 PMC7008637

[B66] Smith W. S. Sung G. Saver J. Budzik R. Duckwiler G. Liebeskind D. S. (2008). Mechanical Thrombectomy for Acute Ischemic Stroke: Final Results of the Multi MERCI Trial. Stroke 39 (4), 1205–1212. 10.1161/STROKEAHA.107.497115 18309168

[B67] Snelling B. Mccarthy D. J. Chen S. Sur S. Elwardany O. Sheinberg D. L. (2019). Extended Window for Stroke Thrombectomy. J. Neurosci. Rural Pract. 10 (2), 294–300. 10.4103/jnrp.jnrp_365_18 31001020 PMC6454953

[B68] Storck T. Schulte S. Hofmann K. Stoffel W. (1992). Structure, Expression, and Functional Analysis of a Na(+)-dependent Glutamate/aspartate Transporter from Rat Brain. Proc. Natl. Acad. Sci. U S A. 89 (22), 10955–10959. 10.1073/pnas.89.22.10955 1279699 PMC50461

[B69] Teichberg V. I. Cohen-Kashi-Malina K. Cooper I. Zlotnik A. (2009). Homeostasis of Glutamate in Brain Fluids: an Accelerated Brain-To-Blood Efflux of Excess Glutamate Is Produced by Blood Glutamate Scavenging and Offers protection from Neuropathologies. Neuroscience 158 (1), 301–308. 10.1016/j.neuroscience.2008.02.075 18423998

[B70] Tutor-Crespo M. J. Hermida J. Tutor J. C. (2004). Activation of Serum Aminotransferases by Pyridoxal-5' -phosphate in Epileptic Patients Treated with Anticonvulsant Drugs. Clin. Biochem. 37 (8), 714–717. 10.1016/j.clinbiochem.2004.03.007 15302618

[B71] Tzingounis A. V. Wadiche J. I. (2007). Glutamate Transporters: Confining Runaway Excitation by Shaping Synaptic Transmission. Nat. Rev. Neurosci. 8, 935–947. 10.1038/nrn2274 17987031

[B72] Vandenberg R. J. Ryan R. M. (2013). Mechanisms of Glutamate Transport. Physiol. Rev. 93 (4), 1621–1657. 10.1152/physrev.00007.2013 24137018

[B73] Verdouw P. D. van den Doel M. A. de Zeeuw S. Duncker D. J. (1998). Animal Models in the Study of Myocardial Ischaemia and Ischaemic Syndromes. Cardiovasc. Res. 39 (1), 121–135. 10.1016/s0008-6363(98)00069-8 9764194

[B74] Verma M. Wills Z. Chu C. T. (2018). Excitatory Dendritic Mitochondrial Calcium Toxicity: Implications for Parkinson's and Other Neurodegenerative Diseases. Front. Neurosci. 12, 523. 10.3389/fnins.2018.00523 30116173 PMC6083050

[B75] Westerhuis L. W. Hafkenscheid J. C. (1983). Apoenzyme Content of Serum Aminotransferases in Relation to Plasma Pyridoxal-5'-Phosphate Concentration. Clin. Chem. 29 (5), 789–792. 10.1093/clinchem/29.5.789 6839455

[B76] WHO (2020). WHO Methods and Data Sources for Global burden of Disease Estimates 2000-2019. Available at: https://cdn.who.int/media/docs/default-source/gho-documents/global-health-estimates/ghe2019_daly-methods.pdf?sfvrsn=31b25009_7 (Accessed December 21, 2021).

[B77] Wu Q. J. Tymianski M. (2018). Targeting NMDA Receptors in Stroke: new hope in Neuroprotection. Mol. Brain 11 (1), 15. 10.1186/s13041-018-0357-8 29534733 PMC5851248

[B78] Yi J. S. Kim T. Y. Kyu Kim D. Koh J. Y. (2007). Systemic Pyruvate Administration Markedly Reduces Infarcts and Motor Deficits in Rat Models of Transient and Permanent Focal Cerebral Ischemia. Neurobiol. Dis. 26, 94–104. 10.1016/j.nbd.2006.12.007 17261368

[B79] Yoo A. J. Pulli B. Gonzalez R. G. (2011). Imaging-based Treatment Selection for Intravenous and Intra-arterial Stroke Therapies: a Comprehensive Review. Expert Rev. Cardiovasc. Ther. 9 (7), 857–876. 10.1586/erc.11.56 21809968 PMC3162247

[B80] Zaghmi A. Dopico-López A. Pérez-Mato M. Iglesias-Rey R. Hervella P. Greschner A. A. (2020). Sustained Blood Glutamate Scavenging Enhances protection in Ischemic Stroke. Commun. Biol. 3 (1), 729. 10.1038/s42003-020-01406-1 33273696 PMC7713697

[B81] Zauner A. Bullock R. Kuta A. J. Woodward J. Young H. F. (1996). Glutamate Release and Cerebral Blood Flow after Severe Human Head Injury. Acta Neurochir Suppl. 67, 40–44. 10.1007/978-3-7091-6894-3_9 8870800

[B82] Zlotnik A. Gruenbaum S. E. Artru A. A. Rozet I. Dubilet M. Tkachov S. (2009). The Neuroprotective Effects of Oxaloacetate in Closed Head Injury in Rats Is Mediated by its Blood Glutamate Scavenging Activity: Evidence from the Use of Maleate. J. Neurosurg. Anesthesiol 21 (3), 235–241. 10.1097/ANA.0b013e3181a2bf0b 19543002

[B83] Zlotnik A. Gurevich B. Cherniavsky E. Tkachov S. Matuzani-Ruban A. Leon A. (2008). The Contribution of the Blood Glutamate Scavenging Activity of Pyruvate to its Neuroprotective Properties in a Rat Model of Closed Head Injury. Neurochem. Res. 33 (6), 1044–1050. 10.1007/s11064-007-9548-x 18080187

[B84] Zlotnik A. Gurevich B. Tkachov S. Maoz I. Shapira Y. Teichberg V. I. (2007). Brain Neuroprotection by Scavenging Blood Glutamate. Exp. Neurol. 203 (1), 213–220. 10.1016/j.expneurol.2006.08.021 17014847

